# Dog breeds and body conformations with predisposition to osteosarcoma in the UK: a case-control study

**DOI:** 10.1186/s40575-021-00100-7

**Published:** 2021-03-10

**Authors:** Grace L. Edmunds, Matthew J. Smalley, Sam Beck, Rachel J. Errington, Sara Gould, Helen Winter, Dave C. Brodbelt, Dan G. O’Neill

**Affiliations:** 1 Bristol Veterinary School, Langford House, Langford, Bristol, BS40 5DU UK; 2 European Cancer Stem Cell Research Institute and School of Biosciences, Cardiff University, Hadyn Ellis Building, Maindy Road, Cardiff, CF24 4HQ UK; 3 VPG Histology (formerly Bridge), Horner Court, 637 Gloucester Road, Horfield, Bristol, BS7 0BJ UK; 4 Division of Cancer and Genetics, School of Medicine, Academic Avenue, Cardiff University, Cardiff, CF62 3LF UK; 5 Langford Veterinary Services, Langford House Langford, Bristol, BS40 5DU UK; 6 Bristol Cancer Institute, Bristol, BS2 8ED UK; 7 Pathobiology and Population Sciences, The Royal Veterinary College, Hawkshead Lane, North Mymms, Hatfield, Herts AL9 7TA UK

**Keywords:** VetCompass, Electronic patient record, EPR, Breed, Dog, Epidemiology, Primary-care, Veterinary, Pedigree, Purebred, Osteosarcoma, Bone tumour, Cancer, Neoplasia

## Abstract

**Background:**

Osteosarcoma is an aggressive and painful bone neoplasm in dogs. Previous studies have reported epidemiological associations suggesting that large body mass, long bone length and the genetics of certain breeds including the Rottweiler are associated with elevated osteosarcoma risk. However, these studies were often limited by selection bias and confounding factors, and have rarely offered insights into breed-associated protection for osteosarcoma. The current study includes 1756 appendicular and axial osteosarcoma cases presenting to VPG Histology (Bristol, UK) compared against a control population of 905,211 dogs without osteosarcoma from primary care electronic patient records in the VetCompass™ dataset.

**Methods and study design:**

Retrospective, case-control study. Multivariable logistic regression analysis explored associations between demographic risk factors (including breed, chondrodystrophy, age, sex/neuter status, skull-shape, and body mass) and osteosarcoma of all anatomical sites.

**Results:**

We identified several breeds with increased and reduced odds of osteosarcoma. At highest risk were the Rottweiler and Great Dane, with > 10 times the odds of osteosarcoma compared with crossbreds, and the Rhodesian Ridgeback, which has not featured in previous lists of at-risk breeds for osteosarcoma, and had an odds ratio of 11.31 (95% confidence interval 7.37–17.35). Breeds at lowest risk of osteosarcoma (protected breeds) included the Bichon Frise, the French Bulldog and the Cavalier King Charles Spaniel, all with odd ratios of less than 0.30 compared with crossbreds. Body mass was strongly associated with osteosarcoma risk; dogs over 40 kg exhibited osteosarcoma odds of 45.44 (95% confidence interval 33.74–61.20) compared with dogs less than 10 kg. Chondrodystrophic breeds had an osteosarcoma odds ratio of 0.13 (95% confidence interval 0.11–0.16) compared with non-chondrodystrophic breeds.

**Conclusions:**

This study provides evidence of strong breed-associated osteosarcoma risk and protection, suggesting a genetic basis for osteosarcoma pathogenesis. It highlights that breeds selected for long legs/large body mass are generally overrepresented amongst at-risk breeds, whilst those selected for short leg length/small body mass are generally protected. These findings could inform genetic studies to identify osteosarcoma risk alleles in canines and humans; as well as increasing awareness amongst veterinarians and owners, resulting in improved breeding practices and clinical management of osteosarcoma in dogs.

**Supplementary Information:**

The online version contains supplementary material available at 10.1186/s40575-021-00100-7.

## Plain English summary

Osteosarcoma is a painful and aggressive bone tumour in dogs that is known to be more common in certain breeds than others. The finding that bone-tumours are more common in certain breeds tells us that a dog’s genetics play a role in bone tumour development. There is much research aimed at identifying exactly which genetic differences cause bone-tumours, and this will allow us to (i) identify which dogs might be at risk and screen them regularly to detect bone-tumours early and (ii) develop new anti-tumour treatments based on genetics.

The current study presents a comparison of bone-tumour risk levels between different dog breeds. Whereas previous studies identified high risk breeds for bone tumours, we also identify those breeds at lowest risk, meaning that the breeds identified here could be compared to identify novel genetic differences which cause bone-tumours. In this study, we also compared various measures of body mass and leg length, and confirmed previous findings that heavier dogs with longer legs are at greatest risk of bone-tumours. This link between the biology of height and the biology of bone-tumours in dogs provides valuable avenues for further study into what causes bone-tumours to develop, and how we might treat them in the future.

## Background

Osteosarcoma is an aggressive bone neoplasm occurring in dogs, which generally presents as lameness or pain associated with a bony or soft tissue mass or swelling [1]. Pathological fracture is reported to occur in 38% of osteosarcoma cases [2, 3]. Treatment for osteosarcoma can include amputation of the affected limb or resection of axial lesions, and adjuvant chemotherapy may be recommended [2, 4, 5]. However, osteosarcoma often undergoes early haematogenous spread and, whilst just 10% of canine osteosarcoma cases present with gross metastases, 90% have been shown to possess microscopic metastatic disease at the time of diagnosis [1, 2, 4, 6]. Therefore whilst amputation is appropriately carried out as palliative surgery to relieve pain, it is unable to prevent the metastatic spread which has already occurred in most osteosarcoma cases, and amputation therefore has little effect on survival. The lungs are the most common site of metastatic spread in canine osteosarcoma, and the median 1 year survival for dogs treated with amputation and chemotherapy is 45–50% [4, 6–8]. Since osteosarcoma causes severe pain, and current therapies offer little possibility of complete clinical cure, canine osteosarcoma represents a significant welfare threat to commonly affected breeds and a source of distress to owners [4, 6].

Radiographically, canine osteosarcoma appears as lytic, proliferative or mixed bone lesions [4]. Osteosarcoma is also categorised by anatomical site. The appendicular skeleton (limbs and pelvis) represents the most common site of disease in large-breed dogs (95% of cases), whereas although only 5% of total osteosarcoma is reported to occur in dogs less than 15 kg, more than 65% of small-breed osteosarcomas are located in the axial skeleton (head, cervical and spinal vertebrae, sternum and ribs) [2, 4, 6]. A study of 85 appendicular osteosarcoma cases reported the most common lesion locations as the proximal humerus (26% of lesions), the distal radius (24%) and the distal tibia (15%), and multiple other analyses support this lesion distribution [1, 2, 4, 9]. It is rare for osteosarcoma to be located as a mid-shaft lesion on any bone or near the elbow [2]. Currently, standard practice is to confirm clinical and imaging diagnoses of osteosarcoma via fine needle aspirate or histopathology. Although pathologists utilise an agreed classification system for histological subtyping of osteosarcoma, there is disputed prognostic utility of the various histological grading schemes available [2, 4].

Several dog breeds are reportedly predisposed to osteosarcoma, including the Rottweiler, Irish Wolfhound, Greyhound and Golden Retriever, and it has been shown that predisposition to osteosarcoma has a genetic basis in dogs [4, 5, 10–13]. The majority of at-risk breeds possess large body mass, and fewer than 5% of osteosarcoma cases are reported in dogs under 15 kg [14]. It is therefore accepted that risk alleles for osteosarcoma may have become concentrated within certain breeds during selection for large body size (or, as some largeness alleles represent the ancestral wolf allele, non-selection for small body size) [10, 13]. Such genetic variants may occur within genes which are the drivers of large body size or, alternatively, they may be variants in genes which do not functionally influence body size but which are inherited in linkage with largeness alleles [10, 13, 15]. Findings from canine Genome Wide Association Studies (GWAS) of osteosarcoma imply that both modes of inheritance may apply for osteosarcoma risk alleles, and such studies have so far identified risk-associated polymorphisms at the Insulin Growth Factor-1 (IGF-1) locus, which is associated with large body size, and at other loci, such as the Cyclin Dependent Kinase CDKN2A/B region [4, 7, 10, 11, 13]. However, only Irish Wolfhounds, Rottweilers and Greyhounds have been included in existing canine osteosarcoma GWAS, meaning that alternative causal variants in other breeds could have been missed while, to-date, protected breeds have been ignored altogether [10, 13, 16]. Overall, the breed-associated genetics of osteosarcoma need to be examined in more detail in order to facilitate the discovery of novel osteosarcoma risk-associated genetic variants [17–19]. Furthermore, the design of previous studies may have resulted in the underestimation of risk in commonly owned breeds, and have not included explicit conformational traits such as leg-length. The identification of novel demographic associations with osteosarcoma will permit the discovery of novel genetic variants, which in turn will enable polygenic risk models to be built, supporting the development of targeted osteosarcoma screening programmes, and permitting breeders to instigate responsible breeding practices, thus improving canine welfare [13]. It will also provide the foundation for further studies to determine whether the variants associated with risk are situated within genes which drive osteosarcoma formation or progression, and therefore whether or not they are potential therapeutic targets [1].

It is likely that additional non-genetic aspects of body size biology, such as epigenetics, along with the environment and nutrition during bone growth also combine with genetic predisposition to initiate osteosarcoma in large-breed individuals. This means that large breed genetics are necessary but not sufficient to induce osteosarcoma. However, non-genetic risk factors for canine osteosarcoma are poorly understood, and the identification of breeds predisposed to, and protected from, osteosarcoma will generate hypotheses for research in this field [16, 20]. In humans, osteosarcoma is rare, affecting 3 individuals per million in the United States each year. However, since it affects adolescents and carries a prognosis of 60 to 70% 5-year survival amongst patients without disseminated disease, and 28% 5-year survival with metastases, osteosarcoma is an important cancer of unmet need [1, 21]. Risk factors for human osteosarcoma appear to parallel those identified in dogs and include large birth weight, early pubertal growth and taller than average height [22]. Studies aiming to predict osteosarcoma risk in humans have been hampered by small clinical sample sizes, and therefore canine studies with larger sample sizes have great potential to inform targeted human analyses which could produce advancements in early detection and intervention [1].

Using anonymised veterinary clinical and demographic data from pathology records originating from VPG Histology [23] and VetCompass™ [24], this study aimed to identify demographic risk factors for canine osteosarcoma with a particular focus on reporting both predispositions and protections associated with breed and conformation [25]. Osteosarcoma of all anatomical sites was included in this study. The primary hypothesis of the current study was that, based on previous studies, the odds of osteosarcoma are higher amongst specific breeds such as Rottweilers, Scottish Deerhounds, Wolfhounds, Greyhounds and Golden Retrievers compared with crossbreds [4, 5, 16]. Furthermore, it was hypothesised that purebred dogs in general have higher odds of osteosarcoma than crossbreds, since crossbred dogs made up only 19.2% of the total osteosarcoma case population in one study [5]. Since reportedly predisposed breeds possess large body mass, a related hypothesis was that heavier weight categories have higher odds of osteosarcoma [2, 5, 10, 13, 26]. Secondary to the breed and body mass hypotheses, we also proposed that dogs with conditions which become inherited during breeding for short leg length, such as chondrodystrophy, would be protected from osteosarcoma compared with non-chondrodystrophic breeds [27–29]. This hypothesis was derived from the observation that human adolescents of greater than median height make up 62% of osteosarcoma cases [30]. A separate hypothesis, unrelated to breed and conformation, was that older dogs have increased odds of osteosarcoma compared with younger animals. An association between age and canine osteosarcoma risk has been reported previously, and ageing is known to increase cancer-risk owing to mechanisms extensively reviewed elsewhere [5, 31, 32].

The current study adds to the existing work on canine osteosarcoma by identifying protected breeds as well as those at-risk. In utilising a large sample size with a representative denominator population, we identified several new breed-associations not previously reported, and eliminated some of the bias associated with previous analyses in the field. Overall, we present novel breed and conformational associations with canine osteosarcoma of all anatomical sites and provide suggestions for avenues of further work.

## Results

### Description of study populations and Univariable logistic regression Modelling

**Fig. 1:**
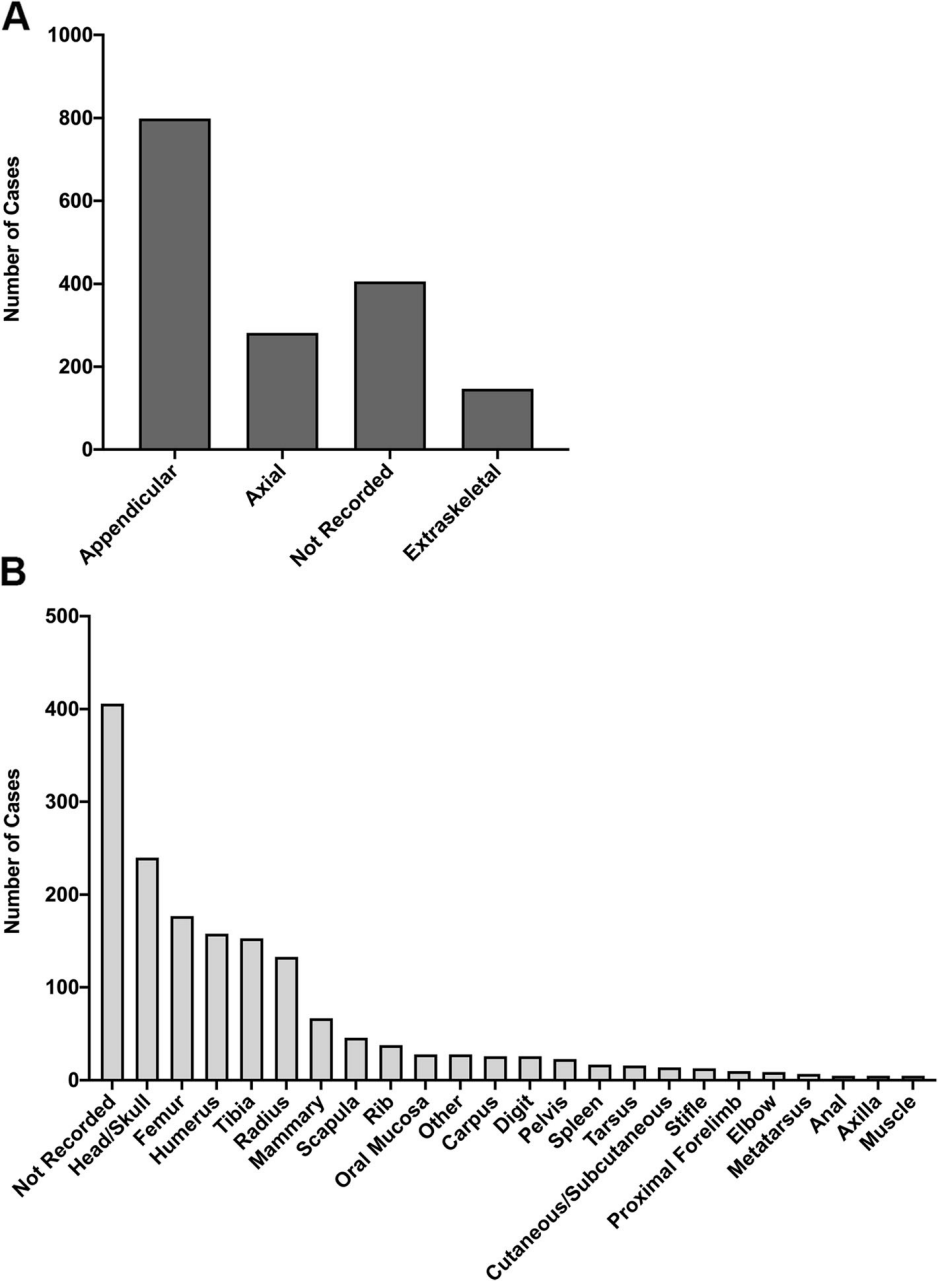
Location distribution for lesions biopsied from 1756 osteosarcoma cases included in the current analysis. **a**. Lesions were categorised as originating from the appendicular skeleton (limbs and pelvis), axial skeleton (head, cervical and spinal vertebrae, sternum and ribs). Extraskeletal osteosarcomas were those not originating from any bony tissue. The lesion location was not recorded for 528/1756 cases. **b.** Osteosarcoma lesions were categorised based on the tissue of origin. Those locations with less than 1 case were grouped as “other” and included 1 renal, 7 hepatic, 3 pulmonary, 1 bladder, 2 gastrointestinal, 1 pleural, 1 thyroid, 1 aortic, 1 jugular groove, 1 lymph node, 1 ocular, 2 fibula, 2 metacarpal and 4 sternal osteosarcomas

The study included 1756 osteosarcoma cases from the VPG Histology dataset and 905,211 controls from the VetCompass dataset. Osteosarcomas were not subdivided by location for analysis; however the distribution of locations included in the case dataset is shown in Fig. 1. Amongst the VPG Histology cases, 45.50% were appendicular (799), 16.00% were axial (282), 8.37% were extraskeletal (147) and 30.06% were of unrecorded location (528 cases).

Of cases, 21.10% (370) were crossbred and 77.40% (1359) were purebred whereas 27.20% (245889) of non-cases were crossbred and 72.4% (655266) were purebred. The remaining cases and controls were of unknown breed (Table 1). The 5 most common breeds amongst cases were Crossbred (*n* = 300, 17.1%), Labrador Retriever (*n* = 260, 14.8%), Rottweiler (*n* = 139, 7.9%), Greyhound (*n* = 103, 5.9%), German Shepherd dog (*n* = 65, 3.7%) and Golden Retriever (*n* = 65, 3.7%). The most common breeds amongst non-cases were Crossbred (*n* = 197,549, 21.8%), Labrador Retriever (*n* = 59,925, 6.6%), Staffordshire Bull Terrier (*n* = 53,934, 6.0%), Jack Russell Terrier (*n* = 48,569, 5.4%) and Cocker Spaniel (*n* = 33,073, 3.7%) (Table 1). Amongst cases, the most common Kennel Club (KC) breed group was Gundog (*n* = 484, 27.6%) whereas amongst non-cases Terriers were the most common group (*n* = 145,828, 16.1%) (Table 1).

**Table 1:** Descriptive statistics and univariable logistic regression results identifying demographic risk factors of osteosarcoma in UK dogs

Variable	***P***-Value for Variable	Category	Case No. (%)	Non-case No. (%)	OR*	95% CI**	***P***-value for Category
Purebred Status	< 0.001	Crossbred	300 (17.1)	197,549 (21.8)	Base		
		Purebred	1429 (81.4)	703,601 (77.7)	1.34	1.18–1.51	< 0.001
		Purebred status unrecorded	27 (1.5)	4061 (0.4)	4.38	2.95–6.5	< 0.001
Breed	< 0.001	Crossbred	300 (17.1)	197,549 (21.8)	Base		
		Rhodesian Ridgeback	29 (1.7)	1495 (0.2)	12.77	8.70–18.76	< 0.001
		Rottweiler	139 (7.9)	7223 (0.8)	12.67	10.35–15.52	< 0.001
		Greyhound (Unspecified)	103 (5.9)	5511 (0.6)	12.31	9.82–15.42	< 0.001
		Great Dane	19 (1.1)	1258 (0.1)	9.95	6.23–15.86	< 0.001
		German Pointer	21 (1.2)	1578 (0.2)	8.76	5.61–13.68	< 0.001
		Mastiff (Unspecified)	35 (2.0)	3096 (0.3)	7.44	5.24–10.58	< 0.001
		Pinscher (Unspecified)	29 (1.7)	2566 (0.3)	7.44	5.07–10.92	< 0.001
		Lurcher	53 (3.0)	6016 (0.7)	5.8	4.33–7.78	< 0.001
		Breed not recorded	27 (1.5)	4061 (0.4)	4.38	2.95–6.50	< 0.001
		Golden Retriever	65 (3.7)	9785 (1.1)	4.37	3.34–5.72	< 0.001
		Collie (Unspecified)	13 (0.7)	1983 (0.2)	4.32	2.47–7.54	< 0.001
		Fox Terrier	5 (0.3)	1013 (0.1)	3.25	1.34–7.88	0.009
		Weimaraner	11 (0.6)	2414 (0.3)	3.00	1.64–5.48	< 0.001
		Standard Poodle	5 (0.3)	1128 (0.1)	2.92	1.20–7.08	0.018
		Labrador Retriever	260 (14.8)	59,925 (6.6)	2.86	2.42–3.37	< 0.001
		Akita (Unspecified)	8 (0.5)	2037 (0.2)	2.59	1.28–5.23	0.008
		Poodle (Unspecified)	4 (0.2)	1050 (0.1)	2.51	0.93–6.74	0.068
		Boxer	35 (2.0)	9438 (1.0)	2.44	1.72–3.47	< 0.001
		Scottish Terrier	5 (0.3)	1455 (0.2)	2.26	0.93–5.48	0.071
		German Shepherd Dog	65 (3.7)	21,360 (2.4)	2.00	1.53–2.62	< 0.001
		Tibetan Terrier	5 (0.3)	1870 (0.2)	1.76	0.73–4.27	0.210
		Hungarian Vizsla	5 (0.3)	1976 (0.2)	1.67	0.69–4.04	0.258
		American Bulldog	8 (0.5)	3225 (0.4)	1.63	0.81–3.3	0.171
		Cairn Terrier	6 (0.3)	2470 (0.3)	1.60	0.71–3.59	0.255
		Dogue de Bordeaux	7 (0.4)	3030 (0.3)	1.52	0.72–3.22	0.273
		Labradoodle	17 (1.0)	7595 (0.8)	1.47	0.90–2.40	0.120
		Alaskan Malamute	4 (0.2)	1915 (0.2)	1.38	0.51–3.69	0.527
		Dalmatian	5 (0.3)	2720 (0.3)	1.21	0.50–2.93	0.672
		English Springer Spaniel	30 (1.7)	20,198 (2.2)	0.98	0.67–1.42	0.908
		Whippet	7 (0.4)	4684 (0.5)	0.98	0.46–2.08	0.967
		Border Collie	29 (1.7)	22,403 (2.5)	0.85	0.58–1.25	0.412
		Bulldog (Unspecified)	4 (0.2)	3232 (0.4)	0.81	0.30–2.19	0.685
		Staffordshire Bull Terrier	63 (3.6)	53,934 (6.0)	0.77	0.59–1.01	0.058
		Other Purebred	223 (12.7)	206,227 (22.8)	0.71	0.60–0.85	< 0.001
		West Highland White Terrier	20 (1.1)	18,875 (2.1)	0.70	0.44–1.10	0.119
		Cocker Spaniel	25 (1.4)	33,073 (3.7)	0.50	0.33–0.75	0.001
		Beagle	6 (0.3)	8070 (0.9)	0.49	0.22–1.10	0.083
		Jack Russell Terrier	33 (1.9)	48,569 (5.4)	0.45	0.31–0.64	< 0.001
		Border Terrier	6 (0.3)	9651 (1.1)	0.41	0.18–0.92	0.030
		Bichon Frise	7 (0.4)	13,268 (1.5)	0.35	0.16–0.74	0.006
		Husky	4 (0.2)	8524 (0.9)	0.31	0.12–0.83	0.020
		Cavalier King Charles Spaniel	7 (0.4)	17,257 (1.9)	0.27	0.13–0.57	0.001
		French Bulldog	4 (0.2)	16,397 (1.8)	0.16	0.06–0.43	< 0.001
		Shar-Pei	NA (NA)	3647 (0.4)			
		Cavachon	NA (NA)	3535 (0.4)			
		Maltese	NA (NA)	3248 (0.4)			
		Cavapoo	NA (NA)	4035 (0.4)			
		Cockapoo	NA (NA)	18,260 (2.0)			
		King Charles Spaniel	NA (NA)	2813 (0.3)			
		Sprocker	NA (NA)	3338 (0.4)			
		Miniature Poodle	NA (NA)	2415 (0.3)			
		Boston Terrier	NA (NA)	1799 (0.2)			
		Chow Chow	NA (NA)	1002 (0.1)			
		Dachshund (Unspecified)	NA (NA)	1692 (0.2)			
		Goldendoodle	NA (NA)	1128 (0.1)			
		Jackapoo	NA (NA)	1362 (0.2)			
		Jug	NA (NA)	1967 (0.2)			
		Miniature Yorkshire Terrier	NA (NA)	1560 (0.2)			
		Puggle	NA (NA)	1173 (0.1)			
		Standard Dachshund	NA (NA)	1133 (0.1)			
KC Breed Group	< 0.001	Toy	26 (1.5)	131,897 (14.6)	Base		
		Gundog	484 (27.6)	135,606 (15.0)	18.11	12.20–26.87	< 0.001
		Hound	164 (9.3)	31,364 (3.5)	26.53	17.54–40.13	< 0.001
		Not KC Recognised	390 (22.2)	263,616 (29.1)	7.51	5.05–11.16	< 0.001
		Pastoral	144 (8.2)	51,675 (5.7)	14.14	9.31–21.47	< 0.001
		Terrier	153 (8.7)	145,828 (16.1)	5.32	3.51–8.07	< 0.001
		Utility	46 (2.6)	102,627 (11.3)	2.27	1.41–3.68	0.001
Body mass (kg)	< 0.001	< 10	89 (5.1)	213,321 (23.6)	Base		
		10–19.9	435 (24.8)	167,774 (18.5)	6.21	4.95–7.81	< 0.001
		20–29.9	392 (22.3)	117,620 (13.0)	7.99	6.35–10.06	< 0.001
		30–39.9	522 (29.7)	69,856 (7.7)	17.91	14.30–22.43	< 0.001
		> 40	231 (13.2)	26,178 (2.9)	21.15	16.56–27.02	< 0.001
		Body mass Unrecorded	87 (5.0)	310,462 (34.3)	0.67	0.50–0.90	0.008
Age (y)	< 0.001	< 3	101 (5.8)	329,270 (36.4)	Base		
		3 to < 6	204 (11.6)	223,344 (24.7)	2.98	2.35–3.78	< 0.001
		6 to < 9	589 (33.5)	162,000 (17.9)	11.85	9.60–14.64	< 0.001
		9 to < 12	589 (33.5)	108,448 (12.0)	17.71	14.34–21.87	< 0.001
		> 12	188 (10.7)	69,726 (7.7)	8.79	6.90–11.20	< 0.001
		Age Unrecorded	85 (4.8)	12,423 (1.4)	22.31	16.71–29.78	< 0.001
Sex/Neuter Status	< 0.001	Female Entire	41 (2.3)	233,772 (25.8)	Base		
		Female Neutered	507 (28.9)	197,768 (21.8)	14.62	10.63–20.10	< 0.001
		Male Entire	59 (3.4)	259,460 (28.7)	1.30	0.87–1.93	0.202
		Male Neutered	574 (32.7)	209,982 (23.2)	15.59	11.35–21.4	< 0.001
		Sex Unrecorded	575 (32.7)	4229 (0.5)	775.24	563.91–1065.77	< 0.001
Dachshund Status	< 0.001	Non-Dachshund-type	1426 (81.2)	696,999 (77.0)	Base		
		Dachshund-type	3 (0.2)	10,288 (1.1)	0.14	0.05–0.44	0.001
		Dachshund Status Unrecorded	327 (18.6)	197,924 (21.9)	0.81	0.72–0.91	< 0.001
Spaniel Status	< 0.001	Non-Spaniel-type	1360 (77.4)	630,349 (69.6)	Base		
		Spaniel-type	69 (3.9)	76,938 (8.5)	0.42	0.33–0.53	< 0.001
		Spaniel Status Unrecorded	327 (18.6)	197,924 (21.9)	0.77	0.68–0.86	< 0.001
Chondrodystrophy Status		Non-Chondrodystriphic	1165 (66.3)	290,490 (32.1)	Base		
		Chondrodystrophic	165 (9.4)	331,858 (36.7)	0.12	0.11–0.15	< 0.001
		Chondrodystrophy Unrecorded	426 (24.3)	282,863 (31.2)	0.38	0.34–0.42	< 0.001
Skull Shape	< 0.001	Mesocephalic	982 (55.9)	452,296 (50.0)	Base		
		Brachycephalic	106 (6.0)	166,883 (18.4)	0.29	0.24–0.36	< 0.001
		Dolichocephalic	341 (19.4)	75,770 (8.4)	2.07	1.83–2.35	< 0.001
		Skull Shape Unrecorded	327 (18.6)	210,262 (23.2)	0.72	0.63–0.81	< 0.001

**Legend: Descriptive statistics and univariable logistic regression results calculated using cases submitted to VPG Histology between 2008 and 2020, and controls enrolled in the VetCompass™ programme during 2016. Percentages represent percentage of the total study population. The breed percentages separated by case and non-case are listed in Supplementary Data Table** **S1**. Results determine associations between demographic risk factors and osteosarcoma diagnosis in UK dogs. *OR Odds ratio **CI **Confidence interval**

Of case dogs, 6.0% (106) were brachycephalic, 19.4% (341) were dolichocephalic, and 55.9% (982) were mesocephalic. Amongst non-cases, 18.4% (166883) were brachycephalic, 8.4% (75770) were dolichocephalic and 50% (452296) were mesocephalic (Table 1). The sex-neuter variable was divided into five categories, of which the most cases were male-neutered (574 dogs, 32.7%) and most non-cases were male-entire (259,460, 28.7%) (Table 1). Chondrodystrophic dogs represented 9.4% of cases (165) whereas amongst non-cases 36.7% (331,858 dogs) were chondrodystrophic (Table 1). All variables assessed in univariable modelling were associated with osteosarcoma with a global variable *p*-value of < 0.05, and were therefore included in multivariable logistic regression modelling (Table 1).

Briefly, univariable regression calculated the odds ratio of osteosarcoma associated with categories (e.g. *Rottweiler*, *< 3 years, male-neutered*) within different variables (e.g. *breed*, *age* and *sex-neuter status*) but did not condition the odds ratios for any variable on the effect of other variables present in the analysis. For example, the effect of different ages on osteosarcoma was not taken into account when considering the effect of different breeds. This analysis was carried out to obtain a global *p*-value for each variable, identifying those strongly associated with an altered risk of osteosarcoma, in order to carry such variables forward into multivariable analysis. A global *p*-value was calculated by comparing the model which contained each variable e.g. *breed*, to a null model without a *breed* variable (but with all the other variables intact), using ANOVA.

In multivariable modelling, the odds ratio of osteosarcoma in each category of the variable e.g. *breed* was calculated conditional on the concurrent effects of the non-breed related factors of *sex-neuter* and *age*. Again, the global effect of each entire variable was then calculated by comparing the model containing the *breed* variable to a model without *breed* but with all other variables intact. In multivariable modelling, the global variable *p*-value is determined using a likelihood ratio test for comparison of multivariable models. Other breed-related variables (which could not be put into the same model as breed, owing to their high correlation with breed) then replaced *breed* in the multivariable model such that *purebred-status, KC breed group, body mass, dachshund status, spaniel status, chondrodystrophy status and skull-shape* were considered one at a time in a model with *age* and *sex-neuter status*. A study-wide significance threshold of *p* <  0.05 was used without adjusting for multiple comparisons since a Bonferroni correction is overly stringent for correlated variables [33].

### Multivariable logistic regression Modelling

#### Breed- related associations

**Fig. 2:**
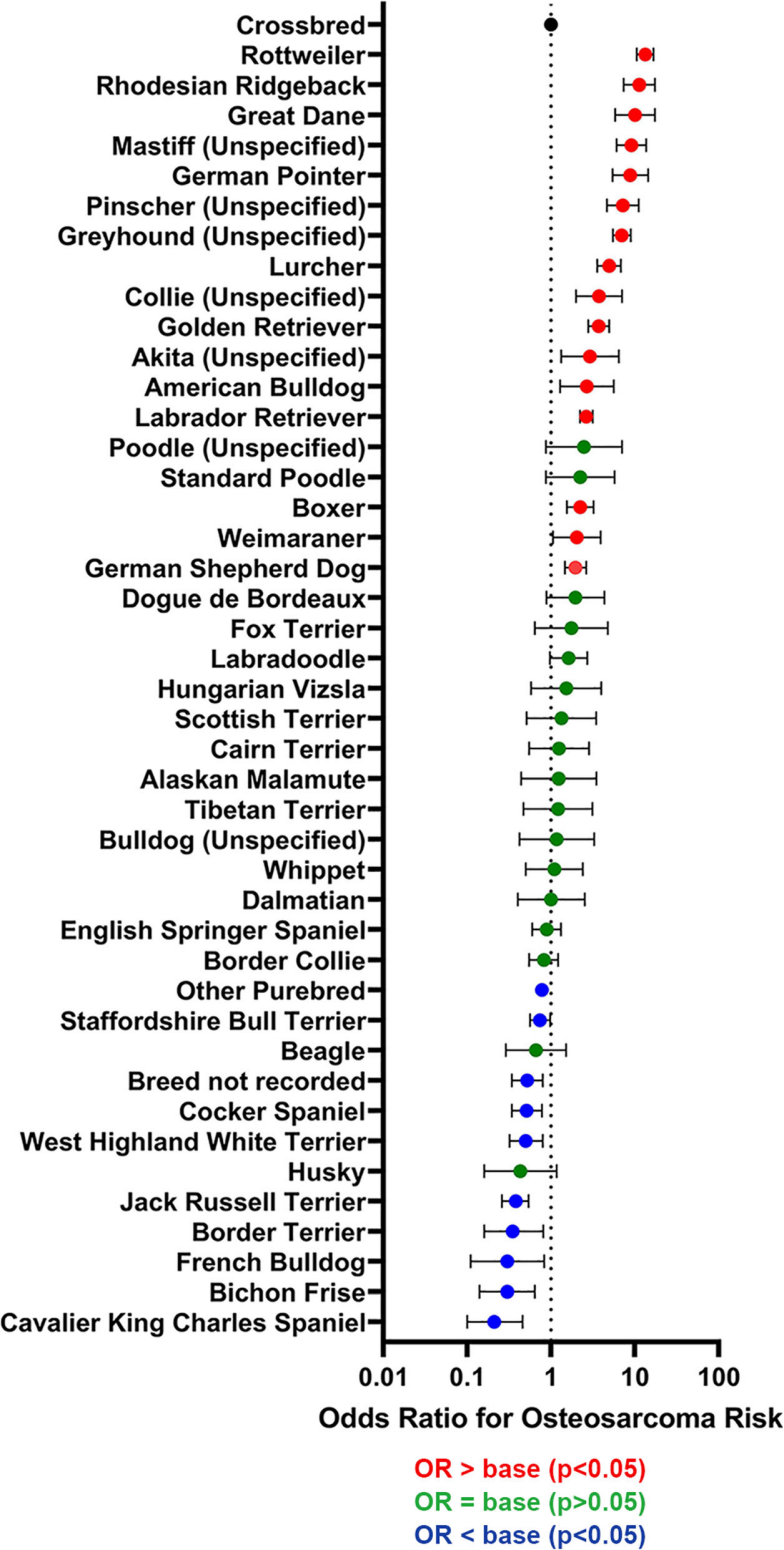
Breed multivariable logistic regression results. Forest plot of Odds Ratios (± 95% confidence intervals) for osteosarcoma risk by dog breed, from multivariable analysis (Table 2) accounting for age and sex/neuter status. Cases were dogs with osteosarcoma confirmed by analysis of biopsies submitted to VPG Histology between 2008 and 2020 and controls were dogs enrolled in the VetCompass™ database during 2016

The final breed model retained breed, age, and sex/neuter status (Fig. 2 and Table 2). The area under the ROC curve was 0.912, indicating a good model fit. After accounting for the effect of the other variables, 27 breeds had increased odds of osteosarcoma compared with crossbred dogs. Breeds with the highest odds ratios (OR) were Rottweiler (OR 13.30, 95% confidence interval (CI) 10.55–16.75), Rhodesian Ridgeback (OR 11.31, 95% CI 7.37–17.35), Great Dane (OR 10.03, 95% CI 5.81–17.32) and Mastiff (OR 9.09, 95% CI 6.06–13.65). The Dalmatian had an OR of 1.00 compared to crossbreds. Thirty breeds had reduced odds of osteosarcoma compared with crossbred dogs. Of these, 16 breeds had zero cases and therefore confidence intervals could not be estimated for the OR of osteosarcoma in these breeds. Of those breeds with at least one case, those with the lowest odds ratios of osteosarcoma included Jack Russell Terrier (OR 0.38, 95% CI 0.26–0.54), Border Terrier (OR 0.35, 95% CI 0.16–0.81), Bichon Frise (OR 0.30, 95% CI 0.14–0.64), French Bulldog (OR 0.30, 95% CI 0.11–0.83) and Cavalier King Charles Spaniel (OR 0.21, 95% CI 0.10–0.46) (Table 2, Fig. 2).

**Table 2:** Multivariable logistic regression results for breed, and non breed-related variables significantly associated with diagnosis of osteosarcoma amongst UK dogs

Variable	Variable ***P***-value	Category	OR	95% CI	Category ***P***-value
Breed	< 0.001	Crossbred	Base		
		Rottweiler	13.30	10.55–16.75	< 0.001
		Rhodesian Ridgeback	11.31	7.37–17.35	< 0.001
		Great Dane	10.03	5.81–17.32	< 0.001
		Mastiff (Unspecified)	9.09	6.06–13.63	< 0.001
		German Pointer	8.84	5.43–14.41	< 0.001
		Pinscher (Unspecified)	7.19	4.65–11.12	< 0.001
		Greyhound (Unspecified)	6.98	5.46–8.93	< 0.001
		Lurcher	4.94	3.57–6.83	< 0.001
		Collie (Unspecified)	3.74	1.99–7.03	< 0.001
		Golden Retriever	3.70	2.77–4.94	< 0.001
		Akita (Unspecified)	2.92	1.32–6.47	0.008
		American Bulldog	2.67	1.28–5.59	0.009
		Labrador Retriever	2.64	2.22–3.15	< 0.001
		Poodle (Unspecified)	2.48	0.87–7.00	0.088
		Boxer	2.23	1.55–3.22	< 0.001
		Standard Poodle	2.23	0.87–5.72	0.096
		Weimaraner	2.03	1.06–3.90	0.034
		German Shepherd Dog	1.96	1.47–2.62	< 0.001
		Dogue de Bordeaux	1.95	0.88–4.33	0.100
		Fox Terrier	1.75	0.64–4.74	0.274
		Labradoodle	1.62	0.97–2.72	0.066
		Hungarian Vizsla	1.52	0.58–3.99	0.395
		Scottish Terrier	1.33	0.51–3.45	0.563
		Cairn Terrier	1.24	0.55–2.84	0.603
		Alaskan Malamute	1.23	0.44–3.47	0.695
		Tibetan Terrier	1.22	0.47–3.12	0.684
		Bulldog (Unspecified)	1.17	0.42–3.27	0.758
		Whippet	1.10	0.50–2.40	0.814
		Dalmatian	1.00	0.40–2.52	0.993
		English Springer Spaniel	0.89	0.60–1.31	0.541
		Border Collie	0.82	0.55–1.22	0.324
		Other Purebred	0.78	0.65–0.93	0.006
		Staffordshire Bull Terrier	0.74	0.56–0.98	0.033
		Beagle	0.66	0.29–1.51	0.328
		Breed not recorded	0.52	0.34–0.80	0.003
		Cocker Spaniel	0.51	0.34–0.78	0.002
		West Highland White Terrier	0.50	0.32–0.80	0.004
		Husky	0.43	0.16–1.17	0.097
		Jack Russell Terrier	0.38	0.26–0.54	< 0.001
		Border Terrier	0.35	0.16–0.81	0.013
		Bichon Frise	0.30	0.14–0.64	0.002
		French Bulldog	0.30	0.11–0.83	0.020
		Cavalier King Charles Spaniel	0.21	0.10–0.46	< 0.001
		Cockapoo	NA (0 cases)		
		Boston Terrier			
		Cavachon			
		Cavapoo			
		Chow Chow			
		Dachshund (Unspecified)			
		Goldendoodle			
		Jackapoo			
		Jug			
		King Charles Spaniel			
		Maltese			
		Miniature Poodle			
		Miniature Yorkshire Terrier			
		Puggle			
		Shar-Pei			
		Sprocker			
		Standard Dachshund			
Sex/Neuter status	< 0.001	Female Entire	Base		
		Female Neutered	7.95	5.78–10.94	< 0.001
		Male Entire	1.17	0.79–1.74	0.441
		Male Neutered	9.39	6.83–12.91	< 0.001
		Sex/Neuter unrecorded	1188.3	855.25–1651.04	< 0.001
Age (Y)	< 0.001	< 3	Base		
		3 to < 6	3.64	2.82–4.70	< 0.001
		6 to < 9	13.30	10.54–16.79	< 0.001
		9 to < 12	18.44	14.59–23.30	< 0.001
		> 12	9.40	7.23–12.23	< 0.001
		Age Unrecorded	2.22	1.62–3.05	< 0.001

**Legend: Full multivariable logistic regression results for variables significantly associated with diagnosis of osteosarcoma amongst UK dogs. The main model included breed-name as the breed variable (**Table 2**), and six breed-related variables** ***(purebred-status, Kennel Club breed group, body mass, dachshund-status, spaniel-status, chondrodystrophy status)*** **then replaced breed-name in the model (**Table 3**). Cases were dogs with osteosarcoma confirmed by analysis of biopsies submitted to VPG Histology between 2008 and 2020 and controls were dogs enrolled in the VetCompass™ database during 2016**

**Table 3:** Multivariable logistic regression results for breed-related variables significantly associated with diagnosis of osteosarcoma amongst UK dogs

Variable	Variable ***P***-value	Category	OR	95% CI	Category ***P***-value
Purebred status	< 0.001	Crossbred	Base		
		Purebred	1.35	1.18–1.54	< 0.001
		Unrecorded	0.52	0.33–0.79	0.003
KC Breed Group	< 0.001	Toy	Base		
		Gundog	14.48	9.72–21.58	< 0.001
		Hound	21.54	14.14–32.81	< 0.001
		Not_KC_Recognised	7.10	4.75–10.59	< 0.001
		Pastoral	11.22	7.33–17.15	< 0.001
		Terrier	3.87	2.54–5.90	< 0.001
		Unrecorded	27.39	18.24–41.13	< 0.001
		Utility	2.42	1.49–3.94	< 0.001
Body mass (kg)	< 0.001	< 10	Base		
		10–19.9	5.91	4.66–7.50	< 0.001
		20–29.9	7.31	5.75–9.30	< 0.001
		30–39.9	15.82	12.49–20.05	< 0.001
		> 40	18.07	13.87–23.53	< 0.001
		Unrecorded	0.15	0.10–0.22	< 0.001
Dachshund status	< 0.001	Non-Dachshund type	Base		
		Dachshund type	0.15	0.05–0.46	0.001
		Unrecorded	0.70	0.61–0.79	< 0.001
Spaniel status	< 0.001	Non-Spaniel type	Base		
		Spaniel type	0.37	0.29–0.47	< 0.001
		Unrecorded	0.65	0.57–0.74	< 0.001
Chondrodystrophy status	< 0.001	Non-chondrodystrophic	Base		
		Chondrodystrophic	0.13	0.11–0.16	< 0.001
		Unrecorded	0.40	0.36–0.45	< 0.001
Skull shape	< 0.001	Mesocephalic	Base		
		Brachycephalic	0.39	0.32–0.48	< 0.001
		Dolichocephalic	1.92	1.68–2.19	< 0.001

**Legend: Full multivariable logistic regression results for variables significantly associated with diagnosis of osteosarcoma amongst UK dogs. The main model included breed-name as the breed variable (**Table 2**), and six breed-related variables** ***(purebred-status, Kennel Club breed group, body mass, dachshund-status, spaniel-status, chondrodystrophy status)*** **then replaced breed-name in the model (**Table 3**). Cases were dogs with osteosarcoma confirmed by analysis of biopsies submitted to VPG Histology between 2008 and 2020 and controls were dogs enrolled in the VetCompass™ database during 2016**

As described in the methods, breed-linked variables (*purebred-status, Kennel Club breed group, body mass, dachshund-status, spaniel-status, chondrodystrophy-status and skull-shape*) individually replaced the *breed* variable in the final multivariable model to evaluate the association of these risk factors with osteosarcoma whilst accounting for other confounding variables (Table 3). Of particular interest were variables relating to both breed and conformation, since the main breed multivariable logistic regression model in the current study showed that many of the predisposed breeds were large breeds whilst many of the protected breeds were small breeds.

**Fig. 3:**
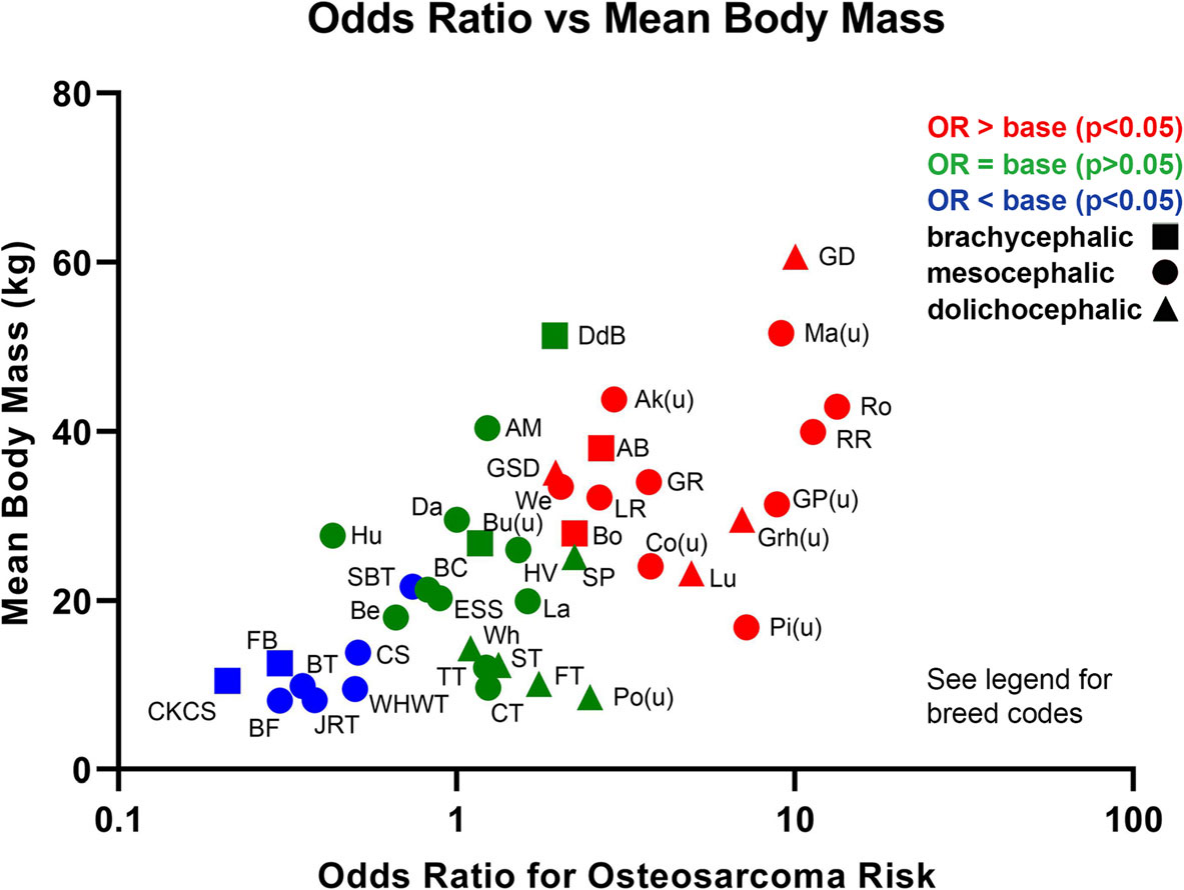
Breed skull shape and OR for osteosarcoma risk (from Table 2 multivariable analysis) plotted against mean body mass. Body mass was calculated as the mean of the VetCompass estimates of average mass for males and females of each breed. Ak(u), Akita (Unspecified); AM, Alaskan Malamute; AB, American Bulldog; Be, Beagle; BF, Bichon Frise; BC, Border Collie; BT, Border Terrier; Bo, Boxer; Bu(u), Bulldog (Unspecified); CT, Cairn Terrier; CKCS, Cavalier King Charles Spaniel; CS, Cocker Spaniel; Co(u), Collie (Unspecified); Da, Dalmatian; DdB, Dogue de Bordeaux; ESS, English Springer Spaniel; FT, Fox Terrier; FB, French Bulldog; GP(u), German Pointer; GSD, German Shepherd Dog; GR, Golden Retriever; GD, Great Dane; Gr(u), Greyhound (Unspecified); HV, Hungarian Vizsla; Hu, Husky; JRT, Jack Russell Terrier; La, Labradoodle; LR, Labrador Retriever; Lu, Lurcher; Ma(u), Mastiff (Unspecified); Pi(u), Pinscher (Unspecified); Po(u), Poodle (Unspecified); RR, Rhodesian Ridgeback; Ro, Rottweiler; ST, Scottish Terrier; SBT, Staffordshire Bull Terrier; SP, Standard Poodle; TT, Tibetan Terrier; We, Weimaraner; WHWT, West Highland White Terrier; Wh, Whippet

When body mass was used in multivariable logistic regression modelling in place of breed, dogs with body mass < 10 kg had the lowest odds of osteosarcoma. The odds of osteosarcoma progressively increased with body size such that dogs > 40 kg had the highest odds of osteosarcoma when compared with < 10 kg (OR 18.07, 95% CI 13.87–23.53). The relationship between body mass and osteosarcoma risk is clearly seen when the OR and mean body mass for each breed are plotted (Fig. 3). Purebred dogs had an OR of 1.25 for osteosarcoma (95% CI 1.11–1.41) compared with crossbred dogs. Dachshund breeds (OR 0.15, 95% CI 0.05–0.46), Spaniel breeds (OR 0.37, 95% CI 0.29–0.47) and chondrodystrophic breeds (OR, 0.13, 95% CI 0.11–0.16) were all associated with reduced risk of osteosarcoma when compared with non-Dachshund, non-Spaniel and non-chondrodystrophic breeds respectively [27–29]. Dolichocephalic dogs (OR 1.92, 95% CI 1.68–2.19) had increased odds of osteosarcoma when compared with mesocephalic dogs, supporting the finding that dogs in the KC hound group, where longer skull-shape predominates, possess the greatest osteosarcoma odds of all KC groups (OR 21.54, 95% CI 14.14–32.81). Of the other Kennel Club breed groups, all showed increased odds of osteosarcoma when compared to the toy breed group. Brachycephalic dogs (OR 0.39, 95% CI 0.32–0.48) were protected when compared with mesocephalics, whereas dolichocephalic dogs were at increased risk compared to mesocephalics, suggesting a linear relationship between nose-length and osteosarcoma risk. This relationship may occur secondary to the generally smaller body mass of brachycephalic breeds, however, it is interesting to note that the heavy Dogue de Bordeaux, which has a brachycephalic skull shape, does not have an elevated osteosarcoma risk comparable to dogs of a similar mass (for example, the Mastiff; Fig. 3).

#### Non-breed related associations

The odds of osteosarcoma were highest amongst dogs 9 to < 12 years (OR 18.44, 95% CI 14.59–23.30) compared with dogs < 3 years old. The sex-neuter category at greatest risk of osteosarcoma was neutered males (OR 9.39, 95% CI 6.83–12.91) compared with entire female dogs [34]. (Table 2).

## Discussion

This study aimed to identify associations between demographic risk factors and osteosarcoma risk amongst UK dogs. A primary focus was placed on breed-related factors in order to facilitate better evidence-based veterinary care, to inform breeding practices, and to generate hypotheses about the genetic basis of osteosarcoma predisposition. Many of the previous studies that examined the epidemiology of canine osteosarcoma were limited because, they utilised entirely secondary care datasets, total study population numbered fewer than 1000 dogs, they often did not have a comparator non-case group, and many were based solely in the USA, where the breed risk of osteosarcoma may differ to the UK [2, 3, 5, 8, 12]. The current study benefitted from inclusion of a large number (1756) of osteosarcoma cases confirmed through analysis of data associated with biopsy samples submitted from veterinary practices to VPG Histology, Bristol, UK [23]. The study additionally benefitted from a control group of 905,211 dogs registered in primary care veterinary practices across the UK within the VetCompass project so that the results could be generalisable to the wider vet-attending dog population [24, 25]. Although univariable and multivariable regression analyses were used to determine the effect of demographic risk factors on the odds of osteosarcoma, only the multivariable results will be discussed because these accounted for the confounding effects of other variables. This study therefore represents one of the largest published studies to estimate the effect of breed-related traits on osteosarcoma in dogs under veterinary care in the UK, and provides important novel information for veterinarians, breeders and researchers.

### Purebred variable

The current study included both purebred and crossbred dogs. We reported higher odds of osteosarcoma amongst purebred dogs compared with crossbreds, which supports the hypothesis that osteosarcoma is a breed-associated disorder.

### Breed variable

After accounting for other variables, 23 breeds with more than 4 cases and more than 1000 controls showed elevated odds of osteosarcoma compared to crossbreds and, of these breeds, the Rottweiler, Rhodesian Ridgeback and Great Dane had over ten times the odds. Although the Rottweiler and Great Dane have been reported as at-risk breeds previously, the current study is the first to identify predisposition for the Rhodesian Ridgeback [2, 4, 5, 10, 12, 13, 26]. Rhodesian Ridgebacks could have been omitted from previous work owing to selection bias, which refers to a scenario in which the composition of the study group differs from the source population, and this biases the association between exposure and outcome [35, 36]. Selection bias exists within studies in which all participants are cases, and in which a control population is not included. Such studies may be unable to distinguish between breeds which represent a high proportion of the caseload of osteosarcoma owing to the popularity of the breed, and those which represent a high proportion of the caseload because the breed is genetically predisposed to osteosarcoma [35]. For example, Rhodesian Ridgebacks are owned by a lower proportion of the general population than Rottweilers (1.7% Rhodesian Ridgeback versus 7.9% Rottweiler ownership within VetCompass), which could have led to case-only studies underestimating the prevalence of osteosarcoma within Rhodesian Ridgebacks, because they present less often to the clinic owing to reduced ownership [5]. Having used a case and a control population, the current study design enabled us to minimize the likelihood of selective sampling, therefore we demonstrated that, as a proportion of Rhodesian Ridgebacks owned, their osteosarcoma risk is actually high [35, 37]. Our results also differ from previous studies which determined that Staffordshire Bull Terriers (SBT) commonly present to veterinary clinics with osteosarcoma, and therefore cited SBT as an at-risk breed [5]. Using a control population of UK owned dogs without osteosarcoma enabled us to show that the presence of SBT in controls relative to cases (SBT are one of the 5 most-owned breeds in the VetCompass control dataset) means that as a proportion of total SBT owned, their osteosarcoma risk is actually small. These findings highlight the requirement for control samples when reporting demographic risk factors of disease [36].

The findings of the current study contrast with one of the largest published analyses of osteosarcoma risk, in which breeds were grouped according to Parker’s genomic classification of dog breeds [5, 38]. In the previous analysis, mastiff-terrier type breeds were shown to have the highest odds of osteosarcoma of all breed groups, however, applying Parker’s classification to the current study shows that the most at-risk breeds (the Rottweiler and the Great Dane) fell into the mountain breed category [5, 38, 39]. The incorporation of age into the breed model in the current study might explain why mountain breeds are shown to be more at risk, since the previous study noted that osteosarcoma occurred at different ages in the different breed groups, but did not include age as a covariate [5]. Furthermore, selection bias is likely to have posed a problem in the previous study, which did not incorporate a control population and used cases in secondary care rather than a mixed primary and secondary care population [5].

Although the current study includes all locations of osteosarcoma, and there are recognised differences in the breed-associations with different locations, the current work does include a spread of appendicular, axial and extraskeletal tumours in order to examine the overall associations between demographic risk factors and osteosarcoma as a whole [1, 9]. Further work will address the location-specific demographic risk factors for osteosarcoma. However, this approach was not taken in the current study because location information was not available for over 500 out of the 1756 cases and excluding these cases would have diminished the power of this analysis.

A novel aspect of the current study was the effort to identify breeds protected from osteosarcoma. It is important to identify protected breeds because their genetics could be compared with the genetics of at-risk breeds to identify allelic variants associated with osteosarcoma risk and protection [1]. Several studies acknowledge that inheritance of osteosarcoma cannot be attributed to a single highly penetrant, large effect genetic variant, but rather adheres to a polygenic risk model associated with inheritance of multiple low penetrance, small effect variants [10]. Improved understanding of such variants and how they influence osteosarcoma risk (both increasing and decreasing) is fundamental for developing osteosarcoma prevention and therapy [1, 13, 16, 20, 40, 41]. In the current study, 30 breeds had reduced odds of osteosarcoma compared with Crossbreds. Of these, 16 had zero cases and therefore, although they were retained in the final model, confidence intervals could not be calculated for the odds of osteosarcoma amongst these breeds. However, given that each of these breeds was represented by at least 1000 dogs in the VetCompass control population, this is highly suggestive of them having reduced osteosarcoma risk. Amongst breeds with at least one case, the Jack Russell Terrier, Border Terrier, Bichon Frise, French Bulldog and Cavalier King Charles Spaniel had the lowest odds of osteosarcoma compared with crossbred dogs. Consistent with our findings that breeds with large body mass are at increased risk of osteosarcoma, the protected breed list comprises breeds of small body mass. Similarly, the Toy KC breed group had the lowest odds of osteosarcoma out of all KC groups. Therefore, the findings of the current study overwhelmingly suggest that protection from osteosarcoma is associated with small body mass. However, despite this finding, there were still some small breeds such as the Scottish Terrier, Cairn Terrier and Whippet that were not protected from osteosarcoma compared to crossbreds. Comparison of the genetics between these small, at- risk breeds against small, protected breeds could point to novel aspects of risk-associated genetic biology for osteosarcoma which occurs independently of body mass.

The effects of chondrodystrophy were analysed as an alternative approach towards exploring associations between body conformation, and osteosarcoma protection. It has been postulated that inheritance of genetic variants predisposing to excessive long bone length and rapid limb growth could underlie the causal biology of osteosarcoma in both humans and dogs. Indeed, in one study, 62% of adolescents with osteosarcoma were shown to be above median height for their age group [22]. Although exact limb length data was not available in the current study, a chondrodystrophy variable was incorporated, as a way of attaining published data about which breeds carry polygenic inheritance of short limb length. The best characterised genetic mutation used as a robust marker of chondrodystrophy is an autosomal dominant fibroblast growth factor (FGF) 4 mutation in chromosome 12 (FGF4L2) that was identified in GWAS of canine limb dysplasia [42]. Breeds in which the FGF4L2 mutation is fixed exhibit a phenotype of extremely short long bones, and intervertebral disc disease [27]. Although 5 other FGF4 retrogenes have been sequenced in dogs, and are also known to affect limb morphology, their frequency of carriage in various canine breeds has not yet been studied therefore chondrodystrophic breeds in the current study were defined as those with published, high-frequency carriage of the FGF4L2 gene [42].

Chondrodystrophic breeds, Spaniel-type breeds and Dachshund-type breeds carry the FGF4L2 mutation at high frequency, and all of these breed-types were associated with protection from osteosarcoma in the current analysis [27–29]. Interestingly, both Cairn and Scottish terriers have been shown to carry chondrodystrophy variants at very low allele frequencies (0 and 0.4 respectively), supporting the inverse relationship we observe between chondrodystrophy gene carriage and osteosarcoma risk, since Cairn and Scottish terriers were not protected from osteosarcoma compared to crossbreds whereas other small breeds were protected [27, 29, 43]. This inference must be made with caution however, as allele frequencies for chondrodystrophy genetic variants have only been calculated using low numbers of animals in these breeds to-date. Nonetheless, the current analysis suggests that small chondrodystrophic dogs may be at lower risk of osteosarcoma compared with small, non-chondrodystrophic dogs, implying that certain routes of breeding for small size, including those related to chondrodystrophy, have resulted in the loss of osteosarcoma risk-associated alleles, whereas others have not [29]. Similarly, brachycephalic dogs were shown to have lower odds of osteosarcoma when compared with mesocephalic or dolichocephalic animals. These results suggest that those individuals with a combination of genetic variants mediating chondrodystrophy or brachycephaly also appear to possess a combination of genetic variants which are associated with protection from osteosarcoma. Previously it was not known whether these two sets of variants were one and the same, however current studies suggest that at least some of those variants which functionally mediate body conformation also functionally affect bone homeostasis and osteosarcoma development.

The presence of an FGF4 retrotransposon associated with appendicular chondrodysplasia also reduces neurocranium size [44, 45], meaning that many chondrodystrophic breeds are also brachycephalic. Furthermore, a missense mutation in bone morphogenetic protein (BMP) 3 is associated with brachycephaly [45, 46] and the presence of a transposable element insertion in SPARK-related modular calcium binding protein (SMOC) 2 gene, which suppresses BMP pathway activity [47, 48], was reported to account for 36% of facial length variation in brachycephalic dogs [44]. Deregulated BMP and FGF signalling are associated with osteosarcoma; and FGF and BMP signalling are known to regulate the development of mesenchymal stem cells to immature and mature osteoblasts, and subsequent skeletal homeostasis [49]. These studies suggest brachycephalic skull shape or chondrodystrophy may both be traits which are markers of reduced activity of signalling pathways, in particular FGF and BMP signalling, which tend to promote osteosarcoma development. Notably, the current study highlighted that Dogue de Bordeaux are a brachycephalic breed in which odds of osteosarcoma are not significantly different to the baseline crossbred population, despite being genetically predisposed to large body mass. Although the reduction in osteosarcoma risk associated with brachycephalic breeds is of scientific interest, selective breeding to enhance brachycephalic traits is not a viable means of reducing canine osteosarcoma risk, since brachycephalic obstructive airway syndrome is a significant welfare concern which is perpetuated by breeding for short skull shape. Furthermore, it is important to emphasise that ‘reduced risk’ does not equate to ‘no risk’ and brachycephalic dogs *can* still get osteosarcoma.

Increasing body mass was shown to be progressively associated with increasing odds of osteosarcoma. An association between large body mass and osteosarcoma risk could occur because allelic variants which mediate osteosarcoma risk are inherited within or along with genetic variants mediating large body size, as discussed above in relation to limb stature. However, it should be remembered that neoplasia is a multifactorial condition, and epigenetic and environmental factors associated with giantism could also underlie the strong association between osteosarcoma risk and large body size in dogs [8, 16, 40, 50]. The results of the current study suggest that larger body size is necessary but not sufficient to produce a high risk (more than ten times the odds of crossbreds) of osteosarcoma, since all breeds in the highly-at risk group have large body mass, however some breeds which attain large body mass, such as the Dogue de Bordeaux and the Alaskan Malamute, have low odds of osteosarcoma. These findings support a mechanism whereby osteosarcoma risk-associated genetics are inherited in some large breeds and absent in others, whilst the environment generated by large-breed biology may also interact with such genetic variants in order to produce osteosarcoma. Larger GWAS comprising both at-risk and protected breeds are required in order to interrogate the genetic determinants of osteosarcoma risk and protection more fully. Whilst some studies have taken the view that breed and body mass are separate variables, in the current study we took body mass to be a breed-associated variable which replaced breed in the final multivariable analysis instead of being included as a covariate. This decision was taken because breed standards specify body mass parameters, therefore genetic variants which mediate body mass are inherently part of each breed, and this concept was supported statistically because if body mass and breed were included in the same model, the VIF score for collinearity carried a *p*-value of < 2.2 × 10^− 16^ suggesting significant multicollinearity between variables [51].

All older age groups had higher odds of osteosarcoma compared with dogs under 3 years of age. However dogs over 12 years old showed lower odds of osteosarcoma than those aged between nine and twelve. Although osteosarcoma reportedly occurs with higher prevalence amongst younger animals when compared to other neoplasms, the current literature suggests that, like most neoplasms, its incidence increases with age, which may be a result of cellular ageing and mutational accumulation [2, 4, 8, 11, 40, 52, 53]. Previous studies have shown a bi-modal distribution of age of onset in osteosarcoma, in which at-risk breeds experience younger age of disease onset [5] and although this was not evident in our analysis of age by categorical variables, when considered as a continuous variable there is a small peak in the number of cases in the second year of life (Supplementary Fig. S7). This parallels the human syndrome whereby genetically at-risk adolescents experience early-onset osteosarcoma, whilst a second population of individuals experience the disease during old-age [54]. Since all osteosarcoma cases are, by definition, cases of neoplasia, further studies utilising a control population of canine patients with any neoplastic lesion, and a case population of osteosarcoma patients, would allow us to determine whether the effects of age seen in the current study are an osteosarcoma-specific effect or are generally applicable to all neoplasms. The current analysis may have underreported the odds of osteosarcoma amongst the oldest dogs for several reasons. Firstly, we and others have shown osteosarcoma to be a cancer of large and giant breed dogs [2, 4, 5, 10]. Large breeds are known to have shorter average lifespans than smaller dogs, therefore nine to 12 years is the age category of highest risk for osteosarcoma in the current analysis, because it reflects the age at death of the high risk breeds for osteosarcoma [55, 56]. Secondly, the current study may be confounded by selection bias, because all cases of osteosarcoma in the current study were diagnosed by biopsy [35]. Elderly veterinary patients may be less likely to receive histopathological analysis of suspected osteosarcoma lesions because the disease is associated with poor prognosis and requires aggressive surgical intervention [1, 2, 5, 12]. Therefore, owners may opt for euthanasia of elderly animals with osteosarcoma more frequently than those with other cancers such as lymphoma, in which less invasive palliative treatment options, such as orally administered chemotherapies, are available [57]. Hence, samples from the over twelve years age group may be underrepresented within the VPG histopathology cases in the current analysis, creating a selection bias. A more detailed analysis of veterinary diagnostic decision making in different tumour settings is required to determine whether this reasoning is valid.

Our results relating to the effects from sex and neutering status supported the current literature that suggests that male animals have increased risk of osteosarcoma compared with females, and that neutered animals of both sexes are more at risk of osteosarcoma compared to their entire counterparts [5, 40, 58]. There is evidence to suggest that reduced levels of circulating gonadal hormones may be associated with increased osteosarcoma risk [58]. However, in the current analysis and in published studies there are many confounding factors that prevent the establishment of a causal role for neutering in osteosarcoma [58, 59]. Importantly, dogs needed to have undergone biopsy for histological analysis to appear in the VPG dataset used in the current study, suggesting a population derived from either insured animals or animals owned by owners who have financed surgical intervention. Data derived by Sánchez-Vizcaíno et al. demonstrated that dogs had significantly increased odds of being neutered if their owners lived in in areas of more affluent socio-economic status according to IMD income deprivation indices (OR 1.90 for male dogs, OR 2.19 for female dogs) [60]. Therefore, neutered animals may be more likely to be owned by owners who are able to finance tumour biopsy, and the socio-economic status of ownership could confound any associations determined between neutering and osteosarcoma risk in the current analysis [60]. Repeating this analysis using osteosarcoma cases from a dataset which allows an even spread of IMD-ranked postcodes to be selected may help to address the role of neutering in osteosarcoma risk [24, 25]. Owner socio-economic status may also have affected the purebred versus crossbred analysis, or indeed the individual breed analysis since the same study also shows that purebred animals are more likely to be owned by more affluent owners than crossbred animals, as are certain breeds, therefore purebreds or breeds favoured by a certain owner demographic could be overrepresented in the VPG Histology caseload [60]. Other experimental and genetic epidemiological methods may be able to interrogate a causal role of gonadal hormone levels in disease amongst canine populations in the future, although owing to the relatively low incidence of osteosarcoma within both canine and human populations, the sample sizes available to such studies are currently too small to ensure adequate experimental power [52].

Further work should consider the differences between appendicular and axial osteosarcoma. Although the various osteosarcoma subtypes are thought to share a common cell of origin, this field is poorly understood, and the demographic risk factors for disease may be different for osteosarcoma when categorised by anatomical location as opposed to osteosarcoma as a whole [61].

An important point to note regarding all of the findings of the current study are that, whilst certain demographic risk factors are associated with protection from osteosarcoma, dogs of small mass, or protected breed, or with brachycephalic confirmation can still get osteosarcoma. Owners and vets should not interpret the findings of the current work to mean that low-risk dogs are completely protected from disease.

## Limitations

In case-control studies, ideally the controls represent the population from which cases are derived. Although in the current analysis this was not fully possible, cases were derived from laboratory samples submitted from primary and secondary care veterinary practices, and controls from VetCompass dogs registered with primary care practices. It was thus considered likely that the VetCompass population was a good estimation of the background veterinary attending population from which the cases originated [62]. However selection bias may have affected the cases whereby only osteosarcoma cases with histologically confirmed diagnoses were included, and these cases may not be selected at random from the true overall UK caseload of canine osteosarcoma cases.

Unmeasured confounding factors may also have influenced the results of the current study because the datasets were acquired over different timescales, and it is not possible to determine the effects of this sampling method on the results obtained. The VetCompass control dataset provides a snapshot of clinic-registered dogs in 2016, whereas the VPG dataset of osteosarcoma cases spans the years 2008–2020. Certain breeds such as brachycephalics and designers had become more popular by 2016, which may make these breeds underrepresented in data from earlier years [63–66]. Thus, such breeds are less likely to feature in the VPG osteosarcoma cases versus the 2016 VetCompass control population, lowering their apparent odds of osteosarcoma. However, if the popularity of these breeds continued to rise between 2016 and 2020, the presence of more recent cases in the VPG osteosarcoma case dataset may offset this effect, since it spans 2008–2020. A more accurate quantification of owned breeds across several years is required to determine the true effect of breed popularity over time on the current study, and repeating the current study using only VetCompass data from 2016 would be advantageous as a comparator for the results presented here. Other factors such as socio-economic status of owners submitting biopsies, and age structure of the breed amongst UK dogs may also have confounded the current study [36].

The current study did not account for the expected breed lifespan when considering demographic risk factors for cancer. However, as alluded to in the discussion of the effect of age on osteosarcoma, various factors such as breed and neuter status may affect the years-at-risk of dogs, and thus certain breeds with longer lifespans, or neutered dogs (shown to live longer on average) may appear to be more at risk of osteosarcoma simply because they experience more years-at risk of disease [67]. The inclusion of this complex variable was beyond the scope of this study because a reliable published lifespan could not be sourced for all of the included breeds, however future analyses considering years-at-risk would be valuable for validation of the conclusions presented here.

Crossbred dogs were used as the comparator for breed-associated risk of osteosarcoma in the current study. This approach uses a precedent established by other studies utilising VetCompass data, whereby the large number of dogs included in the denominator population is considered sufficient to ensure that the crossbred population studied accurately represents the overall crossbred population of UK dogs, and that similarly powered studies could replicate the analysis with a crossbred comparator [68–71]. Crossbreds are a useful comparator for confirmation-related disorder because they comprise variable inheritance of genetic determinants of bodyweight, skull shape and conformation, and they make up the most owned population of the VetCompass denominator dataset. Although crossbreds are often assumed to have hybrid-vigour derived from non-selective breeding, a lack of specificity in breeding practices could also lead to animals perceived as less desirable being used in crosses, making their disease-risk scientifically interesting [68].

Breeds with < 1000 control animals in the current study were combined into a category entitled “Other Purebred”. This variable was associated with a lower risk of osteosarcoma than the crossbred breed category, however combining multiple breeds with varying osteosarcoma risks did not produce an informative result for further research. This strategy was taken to permit the inclusion of all cases in the statistical model. Similarly, breeds with < 4 cases of osteosarcoma or  were also combined into the “Other Purebred” variable, in order to avoid overestimation of odds due to the presence of uncommon breeds with a single case in the dataset. Excluding breeds with low numbers of dogs in this manner did result in the loss of certain breeds from the analysis, including Irish Wolfhounds and Scottish Deerhounds, which previous studies have shown to be predisposed to osteosarcoma. However, estimates of osteosarcoma risk for the full list of breeds with any number of cases are provided in supplementary Table S2, and supplementary Table S1 shows the full case and control population by breed. This information may be of interest to breeders and researchers with a focus on particular uncommon breeds. We also included an unrecorded category for each variable, to ensure that every case appears complete in statistical analysis. This avoids bias induced by omitting incomplete records, since a higher proportion of VPG Histology cases were lacking variable information when compared to VetCompass cases. However, because of this discrepancy in the percentage of unrecorded entries between datasets, the category “variable unrecorded” had altered ORs of osteosarcoma when compared to the base category. Therefore, these results are reported but are not likely to represent hypothesis-generating information and will not be discussed further. Pairwise interactions were not evaluated for all variables in the final models but instead evaluation for interaction was restricted to variables deemed to have a relevant biological interaction (sex and neuter); these variables were combined into one meta-variable to account for interrelatedness.

## Conclusions

This study identifies breed associations with osteosarcoma risk in terms of both predisposition and protection. These results can inform breed health reforms, especially in breeds such as the Rottweiler, Rhodesian Ridgeback and the Great Dane which we have shown to be highly at risk. Other breed-associated variables (such as chondrodystrophy) were associated with protection from osteosarcoma. These findings could be used to identify protection-associated genetic variants for osteosarcoma, for example by identifying variants that are inherited in linkage with chondrodystrophic traits. The findings of this study will also inform research into human osteosarcoma, in which tumour genetics, risk factors including long bone length and body mass, and a clinical presentation involving early metastatic spread have all been shown to parallel canine disease. In summary, the current study generates hypotheses for further work interrogating the genetic and non-genetic risk factors for osteosarcoma, with the aim of informing novel diagnostics and therapeutics for osteosarcoma in both humans and dogs.

## Methods

### Data sources

The study population comprised 1756 osteosarcoma cases and 905,211 non-cases. Cases included all dogs with osteosarcoma in a database of biopsies submitted to VPG Histology (Bristol, UK) between January 2008 and January 2020 inclusive [23–25]. Clinical and demographic information was supplied by the submitting veterinary practice and included an anonymised animal identifier along with breed, date of birth, sex/ neuter status and free-form pathological notes. Additional data fields were completed by a VPG histopathologist to provide the histopathological report, including osteosarcoma diagnosis, date sample received and date of final report. Cases were identified by the presence of the term “osteosarcoma” in the histopathological report. Reports were then reviewed to confirm that histopathological description reached a final diagnosis of osteosarcoma.

The control population included all available dogs under primary veterinary care at clinics participating in the VetCompass programme during 2016, after excluding any dogs with osteosarcoma diagnosis recorded by the veterinarian in the clinical notes. VetCompass collates de-identified electronic patient record data from primary-care veterinary practices in the UK for epidemiological research [24, 25]. Dogs with either a) at least one electronic patient record during 2016 or b) at least one electronic patient record during both 2015 and 2017 were included [72]. Data fields used in the current study included a unique animal identifier along with species, breed, date of birth, sex/neuter status, and body mass, and also free-form text clinical notes, summary diagnosis terms and treatment with relevant dates [24, 25]. Osteosarcoma cases were removed from the overall VetCompass population of dogs using search terms in the clinical notes (osteos*, OSA) to identify candidate cases that were then manually verified to check that an osteosarcoma diagnosis was recorded by the attending veterinarian [36]. As cases were not chosen directly from the VetCompass control population, the incidence of osteosarcoma could not be determined in this study. However, as the control population was selected to represent the wider population of UK dogs that are registered for veterinary care, and therefore to represent the demography of dogs from which cases were sampled, the study design did permit exploration of the demographic risk factors associated with osteosarcoma risk and protection [35, 37, 62].

### Study design

A retrospective, case-control study design was used for risk factor analysis, comparing the VPG osteosarcoma cases and the VetCompass controls [62, 73]. Before commencing the study, a power calculation was conducted based on published works. It was determined that a study with 1756 cases and 905,211 controls would give > 99.99% power to detect differences in the odds of osteosarcoma between the Rottweiler (reported to be the most predisposed breed in previous studies) and crossbreds ( [74] with methodology from [75] Table 6.3). This calculation was based on a previously reported osteosarcoma prevalence of 0.03% amongst crossbreds and 1.14% amongst Rottweilers, with Rottweilers comprising 1.17% of UK dogs [76, 77].

Breed descriptive information recorded in the original VPG and VetCompass datasets was cleaned and mapped to a VetCompass breed list derived and extended from the VeNom Coding breed list [72]. A *purebred status* variable categorised all dogs of recognisable breed as ‘purebred’, including designer crossbreeds with less than two breeds in the cross and with more than 1000 dogs of that designer breed in the VetCompass dataset. All remaining dogs with breed information, including the remaining designer crosses were categorised as ‘crossbred’ [78]. A full list of breed categories derived from the VeNom code is supplied in supplementary Table S3. A separate *breed* variable comprised the individual breed names of dogs listed as ‘purebred’ in the *purebred status* variable, if the breed was represented by over 1000 dogs in the overall study population and by ≥4 osteosarcoma cases. All remaining purebreds were grouped into the “other purebred” category under the *breed* variable. All dogs in the crossbred category of the *purebred status* variable were listed as ‘crossbred’ under the *breed* variable.

Breeds were further characterised by: skull-shape (*dolichocephalic, mesocephalic, brachycephalic, unrecorded*); spaniel-status (*spaniel, non-spaniel, unrecorded*) and dachshund-status (*dachshund, non-dachshund, unrecorded*) for analysis. A chondrodystrophic variable categorised pure-bred dogs as chondrodystrophic where there was published evidence that the allele encoding an autosomal dominant FGF4 mutation for chondrodystrophy located on chromosome 12 was fixed in the breed population [27–29]. A table of breeds included in these lists are provided in supplementary Table S4. Where breeds could not be classified owing to a lack of published information, they were denoted as unrecorded. A *Kennel Club breed group* variable classified breeds recognised by the UK Kennel Club (KC) into their relevant breed groups (Gundog, Hound, Pastoral, Terrier, Toy, Utility and Working) and all remaining types were classified as non-Kennel Club recognised [79]. Toy breeds were used as the base for KC breed group analysis since they possess the smallest body mass of all groups, facilitating assessment of breed and confirmation related hypotheses.

Neuter status was defined by the final available electronic patient record value in each dataset. Sex and neuter were combined into one variable after showing high collinearity during modelling [51]. Adult body mass was not available for VPG Histology cases. Therefore body mass was imputed for the VPG Histology dataset based on VetCompass standard weights for breed/sex combinations. These standards were calculated as the mean of all body mass (kg) values recorded for all dogs older than 18 months within each breed/sex combination in VC, where 100 dogs of that breed were available. Body mass (kg) values for both datasets were then categorised: < 10.0, 10.0 to < 20.0, 20.0 to < 30.0, 30.0 to < 40.0 and ≥ 40.0. Age was defined at the date of histological submission for the VPG cases [24, 72, 80] and on December 31, 2016 for the VetCompass non-cases. Age (years) was categorised as: ≤ 1.0, 1.0 to < 3.0, 3.0 to < 6.0, 6.0 to < 9.0, 9.0 to < 12.0 and ≥ 12.0. Where mean breed bodyweight was plotted against osteosarcoma risk, if multiple VeNom breed terms were included in the Final Breed Term used to determine risk (e.g. ‘Akita – unspecified’ includes dogs recorded as Akita, American Akita and Japanese Akita, see supplementary data S2) then, where available, the mean weights of males and females of all the breeds included was utilised.

### Statistical analysis

Following internal validity checking and data cleaning in Excel (Microsoft Office Excel 2013, Microsoft Corp.), data were cleaned in Rstudio™ using the following packages: plyr, dplyr, data.table, tidyR, and stringr [81–84]. Binary logistic regression modelling was executed using the glm-logit function in the R-stats package to determine univariable associations between risk factors (*purebred-status, breed, Kennel Club breed group, body mass, age, sex/neuter, dachshund-status, spaniel-status, chondrodystrophy-status and skull-shape*) and osteosarcoma [85]. Univariable evaluation showed that the median age of cases (8.50 years, IQR 6.58–10.50) was higher than non-cases (4.40 years, IQR 1.87–8.08) (Mann-Whitney test *p* <  0.001). The median adult body mass amongst cases (29.78 kg, IQR 18.51–35.74) was higher than non-cases (16.29 kg, IQR 8.95–21.95) (Mann-Whitney test, *p* < 0.001) (Table 1).

Because breed was a factor of primary interest for the study, variables derived from the breed information were tested for collinearity using a VIF score available in the caret package [51, 86–89]. Variables that were highly collinear with breed *(purebred, Kennel Club breed group, body mass*, *dachshund-status, spaniel-status, chondrodystrophic, skull-shape*) were excluded from initial breed multivariable modelling. VIF scores for the breed multivariable model are included in supplementary Table S5 to show that no significant collinearity remained after taking this approach. Instead, each of these variables individually replaced the *breed* variable in the final breed-focused model to evaluate their effects after taking account of the other variables. Risk factors with liberal associations in univariable modelling (*P* < 0.02) were taken forward for multivariable evaluation [90]. The area under the ROC curve was calculated using the pROC package and used to evaluate the quality of the model fit and discrimination (non-random effect model) [90]. No observations were dropped from the model during fitting, meaning that confidence intervals and *p*-values were generated for breeds with no cases, although these are not reported. Statistical significance was set at *P* < 0.05 [91]. A global *P*-value for each variable was calculated for the univariable models using ANOVA and for multivariable models using the likelihood ratio test available in the package lmtest [92]. The R script used to execute the above analyses is available at https://github.com/ge8793/Osteosarcoma_Public_Data .

## Supplementary Information

**Additional file 1: Table 1** from the main article in excel format. **Table 2** from the article in excel format. **Table 3** from the article in excel format. **Supplementary Data S1** – cases and non-cases split by breed. **Supplementary Data S2** - results from multivariable main breed model with all breeds included if associated with at least 1 case. **Supplementary Data S3** - full list of breed terms derived from VeNom terms. **Supplementary Data S4** - List of chondrodystrophic breeds. **Supplementary Data S5** - VIF scores and area under the ROC curve. **Supplementary Data S6** – List of breeds by skull shape.

**Additional file 2: Supplementary Fig. S7** – Age distribution of the VPG osteosarcoma case dataset.

## Data Availability

The datasets generated and/or analysed during the current study are available in the Royal Veterinary College repository at http://researchonline.rvc.ac.uk/id/eprint/13028

## Abbreviations

BMP: Bone morphogenetic protein
CI: Confidence interval
FGF: Fibroblast growth factor
GWAS: Genome wide association study
IQR: Interquartile range
KC: The Kennel Club
OR: Odds ratio
RVC: Royal Veterinary College
SMOC: SPARC related modular calcium binding 1
