# Supplementary material for: Dog breeds and body conformations with predisposition to osteosarcoma in the UK: a case-control study
Source: Canine Med Genet. 2021 Mar 10;8:2. doi: 10.1186/s40575-021-00100-7 (PMC7944903; doi:10.1186/s40575-021-00100-7)

**Supplementary Figure S7**

Age distribution of 1623 cases of canine osteosarcoma from the VPG dataset (excluding cases for which no age information was available). The number of cases are plotted by the year of life in which the cases occurred (i.e. ‘7’ indicates the number of cases in animals from 6 years and 1 month up to 7 years and 0 months of age). Numbers of cases at each age are indicated above the bars. Note for animals in their second year of life (13 – 24 months) there is a spike in cases (arrow).


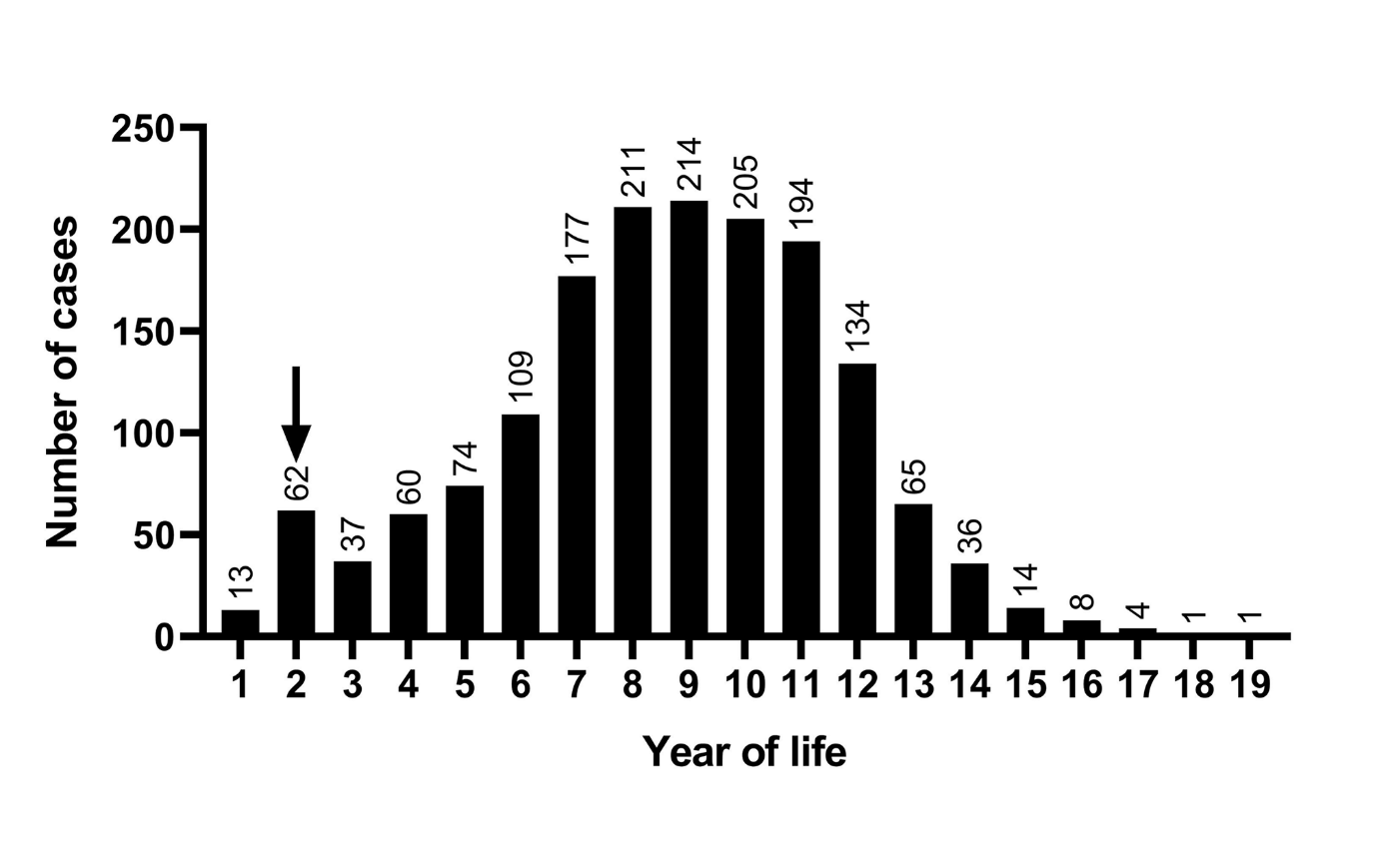

Supplement: Supplementary file 2 — Additional file 2: Supplementary Fig. S7 – Age distribution of the VPG osteosarcoma case dataset. [file 40575_2021_100_MOESM2_ESM.docx]
